# Frailty and medication adherence among older adult patients with hypertension: a moderated mediation model

**DOI:** 10.3389/fpubh.2023.1283416

**Published:** 2023-12-05

**Authors:** Anshi Wang, Jingjing Wan, Lijun Zhu, Weiwei Chang, Liying Wen, Xiubin Tao, Yuelong Jin

**Affiliations:** ^1^School of Public Health, Wannan Medical College, Wuhu, China; ^2^Institutes of Brain Science, Wannan Medical College, Wuhu, China; ^3^Department of Nursing, Anhui College of Traditional Chinese Medicine, Wuhu, China; ^4^Nursing Department, Yijishan Hospital of Wannan Medical College, Wuhu, China

**Keywords:** older adult patients, hypertension, frailty, health literacy, medication adherence, educational level

## Abstract

**Objective:**

Medication adherence has a critical impact on the well-being of older adult patients with hypertension. As such, the current study aimed to investigate the mediating role of health literacy between frailty and medication adherence and the moderating role of educational level.

**Methods:**

This cross-sectional study included patients admitted to the geriatric unit of a hospital. Participants were interviewed using the four-item Morisky Medication Adherence Scale, the Frailty Phenotype Scale, and the Health Literacy Management Scale. Spearman’s correlation coefficients were used to assess the association between variables. Mediation and moderated mediation analyses were performed using Process version 4.1 via Model 4 and 14, respectively.

**Results:**

Data from 388 participants were analyzed. The median (IQR [P_25_–P_75_]) score for medication adherence was 4.00 (2.00–4.00). Results revealed that after controlling for age, sex, hypertension complication(s) and body mass index, frailty significantly contributed to medication adherence (β_total_ −0.236 [95% confidence interval (CI) −0.333 to −0.140]). Medication adherence was influenced by frailty (β_direct_ −0.192 [95% CI −0.284 to −0.099]) both directly and indirectly through health literacy (β_indirect_ −0.044 [95% CI −0.077 to −0.014]). Educational level moderated the pathway mediated by health literacy; more specifically, the conditional indirect effect between frailty and medication adherence was significant among older adult hypertensive patients with low, intermediate, and high educational levels (effect −0.052 [95% CI −0.092 to −0.106]; effect −0.041 [95% CI −0.071 to −0.012]; effect −0.026 [95% CI −0.051 to −0.006]). The relationship between frailty and medication adherence in older adult patients with hypertension was found to have mediating and moderating effects.

**Conclusion:**

A moderated mediation model was proposed to investigate the effect of frailty on medication adherence. It was effective in strengthening medication adherence by improving health literacy and reducing frailty. More attention needs to be devoted to older adult patients with hypertension and low educational levels.

## Introduction

The burden of disease due to hypertension remains high because it is the most important risk factor for all-cause mortality, disability, and global disability-adjusted life years (1). The prevalence of hypertension is significantly higher among older vs. younger individuals (2, 3). A pooled analysis of 104 million subjects found that, between 1990 and 2019, the prevalence and number of individuals with hypertension 30–79 years of age changed from 32% among females and 34% in males to 32% in females and 32% in males, and from 648 million to 1,278 million, respectively (4). A relatively recent national survey conducted in 2018 revealed that the weighted prevalence of hypertension among Chinese adults was 27.5 (5). The prevalence of hypertension in rural adults was higher than that in urban adults (29.4% vs. 25.7%, respectively) (6). With an increase in age among populations, the prevalence of hypertension has increased significantly, with >50% in 60–69 years and >60% in those ≥70 years of age (6). Studies have shown that most patients with hypertension worldwide do not meet blood pressure control standards, especially in low-income countries (4, 7, 8). Poor medication adherence is also a key factor (9). The prevalence of hypertension can be decreased in settings with a range of income levels by improving treatment and control, although there are significant differences in the rates of hypertension treatment and control across nations (4). We cannot reverse the trend in aging populations, which poses a serious threat to the burden of hypertension. However, measures can be taken to mitigate this burden, such as improving patient care and management and, specifically, increasing drug adherence.

Medication adherence refers to the consistency between patient medical adherence and physician recommendations (10). It is influenced by factors including patient-provider relationships, health literacy, age, sex, education, place of residence, income, frailty, family support, attitude, cost of medication, and comorbidities (11–14). Poor medication adherence is the leading cause of poor blood pressure control (15, 16). In one study, patients with good medication adherence were 45% more likely to achieve blood pressure control than those with poor adherence (17), and good medication adherence reduced the risk for cardiovascular outcomes by 37% (18). According to one study, more than one-third of cardiovascular fatalities among Chinese individuals are attributable to poor blood pressure management (19). Improved medication adherence is believed to have a substantially more significant impact on health than improving specific medical treatments/therapies (20), and enhanced medication adherence can significantly reduce cardiovascular events (21). In addition, according to the World Health Organization, poor adherence is the primary factor responsible for uncontrolled blood pressure (22). Therefore, increasing medication adherence in patients with hypertension is essential.

Frailty is defined as a reduction in physiological capacity and increase in vulnerability to stress, both of which are common in older individuals (23). At ages >60 and 80 years’, the prevalence of frailty increases to 15–20 and 30%, respectively (24). This has many similarities to individuals who are susceptible to hypertension. Frailty is an important determinant of longevity and quality of life among the older adult (25) and can be reversed, especially if detected early (26). A population-based study in China reported that the prevalence of frailty in older adults with hypertension was 13.8% (27). Hypertension is more likely to occur in fragile, older adult individuals (28). Frailty negatively affects blood pressure control in older adult patients with hypertension (29) as well as prognosis and adherence to therapy (11). The physical and mental health of older adult individuals are negatively affected due to the onset and development of frailty, which is frequently accompanied by several medical and mental illnesses such as anxiety, depression, cardiovascular disease, and cognitive impairment. Early recognition of frailty in older adult patients with hypertension and appropriate counseling will help ensure their quality of life (11). A possible explanation for this is that the degeneration of physiological functions in the older adult accelerates the progression of frailty, and the occurrence of frailty leads to further deterioration of physiological functions, resulting in the abnormal distribution and metabolism of drugs in the body, eventually increasing the occurrence of adverse drug reactions (30). In addition, frail patients tend to experience negative emotions that exacerbate adverse drug effects and may choose avoidance behaviors that translate into low medication compliance (30). Several studies have concluded that patients with frailty and chronic diseases exhibit poor medication adherence (24, 31, 32).

Health literacy refers to the ability of individuals to acquire, understand and use basic health information and health services so they are able to make the correct health decisions to promote and maintain their own health (33). It can also be condensed into the capacity to make decisions that would improve and preserve one’s well-being (34). Studies have shown that patient health literacy levels increase with educational level (35–37). Health literacy plays a significant role in facilitating the management and prevention of various chronic diseases, reducing the occurrence of various complications, and improving quality of life (38). Since the concept of health literacy was proposed, an increasing number of studies have addressed its relationship with medication adherence (39–42). High health literacy may enhance patient capacity for self-management and medication adherence (43). Studies have presented conflicting results regarding the association between medication adherence and health literacy, including a significant positive correlation (44–46), a significant negative correlation (47), and no significant correlation (48–50). Meanwhile, inadequate health literacy is a concern because it directly influences patient decisions to take medications (51). Compared with individuals with sufficient health literacy, inadequate health literacy has been associated with less optimal medication adherence (52, 53). Patients with low health literacy are more likely to misinterpret prescription label information, which can lead to adverse effects such as poor medication adherence (54). Conversely, better medication adherence and decreased likelihood of misinterpreting pharmaceutical recommendations have been associated with higher levels of health literacy (55). In addition, some studies have demonstrated an association between frailty and the absence of health literacy (56, 57). The association between adequate health literacy and a lower frequency of frailty was also supported by a recent study conducted in 2023 among older community-dwelling adults (58).

In clinical practice, it is crucial to understand the potential mechanisms underlying these predictions and how they relate to medication adherence. In summary, the primary objective of current study was to determine the impact of frailty on medication adherence in older adult patients with hypertension; the second objective was to verify whether health literacy mediates the relationship between frailty and medication adherence; and the third objective was to assess the level of education as a moderating variable. The present study was prompted by lack of studies investigating the impact of frailty on medication adherence and health literacy. We established and verified a moderated mediation model based on two additional variables (i.e., health literacy and educational level). Therefore, our first hypothesis proposes that the association between frailty and medication adherence is significantly mediated by health literacy; the second proposes that the association between health literacy and medication adherence is moderated by educational level.

## Materials and methods

### Study design

A cross-sectional analytical observational design was used to explore the influence of frailty and health literacy on medication adherence among older individuals with hypertension, from June 1, 2019, to December 31, 2019, among geriatric inpatients at a hospital in Wuhu, China. Data were obtained using a questionnaire. This study was approved by the Institutional Research Ethics Committee, and informed consent was obtained from all subjects included in the study. Inpatients ≥60 years of age, who had already been diagnosed with hypertension, were recruited for this study. Individuals who were unwilling to participate in the study and could not express themselves clearly were excluded. Patients who fulfilled the inclusion criteria were selected daily through the admission system and face to face interviewed the next day, after which informed consent was obtained. Each Saturday, a questionnaire was administered to the recruited subjects. In total, 400 patients who fulfilled the criteria were initially recruited, 10 of whom refused to complete the questionnaire on Saturday, and 2 had already been discharged from hospital before completing the questionnaire. Ultimately, therefore, the final sample comprised 388 subjects, corresponding to a response rate of 97%.

### Measures

Trained personnel were recruited to distribute and collect the paper questionnaires, explain the items to illiterate individuals, and record the questionnaire. The questionnaire recorded sociodemographic characteristics such as age, sex, smoking status, alcohol consumption status, height, weight, and educational level, as well as three specific scales.

Medication adherence was evaluated using the 4-item Morisky Medication Adherence Scale (MMAS-4), a reliable self-reported scale (59). Each item was assigned a score of 0 or 1 for “yes” or “no,” respectively. The total score ranged from 0 to 4, with higher scores indicating better medication adherence (45). The Cronbach’s α in the current study was 0.832, demonstrating the reliability of internal consistency.

Frailty was measured using the Frailty Phenotype Scale (FPS), developed by Fried and colleagues in 2001 (60) and widely used in clinical research (61). The Frailty Phenotype Scale was included in the Chinese Expert Consensus for Assessment and Intervention in Older Adult Patients with Frailty (62) and is also widely used by Chinese researchers (63–65). The Frailty Phenotype Scale consists of 5 items addressing unintentional weight loss, walking speed, grip strength, self-reported exhaustion, and physical activity. Each item was assigned a score of 0 or 1 corresponding to a response of “no” or “yes,” respectively. The cumulative score ranged from 0 to 5, with a higher score indicating a higher degree of frailty (66). The Cronbach’s α in the present study was 0.749, demonstrating the reliability of internal consistency.

Health literacy was evaluated using the Health Literacy Management Scale (HeLMS) proposed by Jordan et al. (67). The Chinese version of the HeLMS was adapted according to Chinese cultural customs and population characteristics to assess health literacy in patients with chronic diseases, especially hypertension (68). It comprises 24 items. Each item was scored on a five-point Likert scale (from totally unable or unwilling to without any difficulty or great willingness), with a total score of 120. A higher score indicated a higher level of health literacy. The Cronbach’s α in the current study was 0.917, demonstrating the reliability of internal consistency.

### Statistical analysis

All statistical analyses were performed using SPSS version 25.0. Subject sociodemographic and clinical characteristics and study variables are described. Categorical variables are described as number (percentage), and numerical variables are expressed as mean with standard deviation (SD) or median (interquartile range [i.e., P_25_, P_75_]). The study variables were confirmed to have skewed distributions according to the Kolmogorov–Smirnov test, including frailty, health literacy, and medication adherence. Spearman’s correlation coefficients were used to assess associations among educational level, frailty, health literacy, and medication adherence. To statistically test for the presence of variance in the common methods, the Harman single factor test in SPSS v.25.0 and Confirmatory Factor Analysis in Mplus version 8.0 were performed. In addition, mediation analysis was performed to examine whether health literacy mediated the relationship between frailty and medication adherence (PROCESS 4.1, Model 4). Finally, a moderated mediation analysis was performed using PROCESS 4.1 (Model 14) to examine whether educational level moderated the relationship between health literacy and medication adherence. To confirm the moderating role of education level on health literacy and medication adherence, a simple slope test was performed to assess the relationship between health literacy and medication adherence at different educational levels. The indirect effect of mediation was estimated using a 5,000-sample bootstrapping method, with a significant effect indicated by a 95% confidence interval (CI) that did not include zero. Differences with *p* < 0.05 were considered to be statically significant.

## Results

### Common-method variance test

The results of the Harman single-factor test revealed that 6 factors had Eigenvalues >1, and the first common factor explained 26.71% (<40%) of total variation. The fitting indices of the confirmatory factor analysis were poor: root mean square error of approximation (i.e., “RMSEA”) = 0.200; comparative fit index (i.e., “CFI”) = 0.342; Tucker–Lewis index (i.e., “TLI”) = 0.298; and standardized root mean square residual (i.e., “SRMR”) = 0.237. Therefore, variance in the common method was not a concern.

### Patient characteristics

Subjects exhibited a mean (± SD) body mass index of 23.96 ± 2.93 kg/m^2^; 190 patients were male (49.0%), 47 had a high school diploma or higher (12.1%), and 119 (30.7%) were 65–70 years of age (Table 1). Based on diagnosis, 137 subjects developed hypertensive complications. In addition, the median (IQR [P_25_–P_75_]) scores for medication adherence, frailty and health literacy were 4.00 (2.00–4.00), 1.00 (0.00–2.00) and 78.00 (70.00–90.00), respectively.

**Table 1:** Participants’ characteristics (*n* = 388).

	*N* or mean/median	% or SD or (*P25*, *P75*)
**Socio-demographic characteristics**		
**Age**		
60~	88	22.7
65~	119	30.7
70~	11	2.8
75~	112	28.9
≥80	58	14.9
**Gender**		
Male	190	49.0
Female	198	51.0
**Educational level**		
Primary and below	220	56.7
Junior middle school	121	31.2
High school	36	9.3
Junior college and above	11	2.8
BMI	23.96	2.93
**Clinical characteristic**		
**Hypertension complication**		
Yes	137	35.3
No	251	64.7
**Study variables**		
Medication adherence	4.00	(2.00, 4.00)
Frailty	1.00	(0.00, 2.00)
Health literacy	78.00	(70.00, 90.00)

### Correlations among variables

The results of correlation analyses are summarized in Table 2. Medication adherence (*r_s_* = 0.196, *p* < 0.001), frailty (*r_s_* = −0.117, *p* < 0.05) and health literacy (*r_s_* = 0.354, *p* < 0.001) are all significantly correlated with educational level. Frailty (*r_s_* = −0.223, *p* < 0.001) and health literacy (*r_s_* = 0.301, *p* < 0.001) were both significantly correlated with medication adherence. In addition, a significant and negative correlation was found between frailty and health literacy (*r_s_* = −0.128, *p* < 0.05).

**Table 2:** Correlations between educational level, medication adherence, frailty and health literacy.

	Educational level	Medication adherence	Frailty	Health literacy
Educational level	1.000			
Medication adherence	0.196^***^	1.000		
Frailty	−0.117^*^	−0.223^***^	1.000	
Health literacy	0.354^***^	0.301^***^	−0.128^*^	1.000

^*^p < 0.05, ^**^p < 0.01, ^***^p < 0.001.

### Testing for mediation

The mediating effects of health literacy are summarized in Table 3. Frailty exerted a significant negative effect on medication adherence (β = −0.236, *t* = −4.809, *p* < 0.001) and on health literacy (β = −1.583, *t* = −2.832, *p* < 0.01). When the mediating variable health literacy was added, the direct effect of frailty on medication adherence remained significant (β = −0.192, *t* = −4.072, *p* < 0.001), indicating partial mediation. Health literacy had a significant positive effect on medication adherence (β = 0.028, *t* = 6.583, *p* < 0.001). The partial mediation of health literacy was verified using the bootstrapping method, and the indirect effect accounted for 18.64% of the total effect (Table 4). Thus, this model indicated that the first hypothesis was fulfilled. More specifically, the relationship between frailty and medication adherence was partially mediated by health literacy, and frailty was associated with medication adherence both directly and indirectly through health literacy.

**Table 3:** Testing the mediation effect of frailty on medication adherence through health literacy.

	Model 1 (medication adherence)		Model 2 (health literacy)		Model 3 (medication adherence)	
	*β*	*t*	*β*	*t*	*β*	*t*
Frailty	−0.236	−4.809^***^	−1.583	−2.833^**^	−0.192	−4.072^***^
Health literacy					0.028	6.583^***^
*R* ^2^	0.090		0.061		0.183	
*F*	7.574^***^		4.924^***^		14.235^***^	

Adjusted age, gender, BMI and hypertension complication.

^*^p < 0.05, ^**^p < 0.01, ^***^p < 0.001.

**Table 4:** Mediation model effects.

	*β*	Se	*t*	95% CI	
Total effect	−0.236	0.049	−4.809***	−0.333	−0.140
Direct effect	−0.192	0.047	−4.072^***^	−0.284	−0.099
Indirect effect	−0.044	0.016	—	−0.077	−0.014

Adjusted age, gender, BMI and hypertension complication.

^*^p < 0.05, ^**^p < 0.01, ^***^p < 0.001.

### Testing for moderated mediation

**Figure 1:**
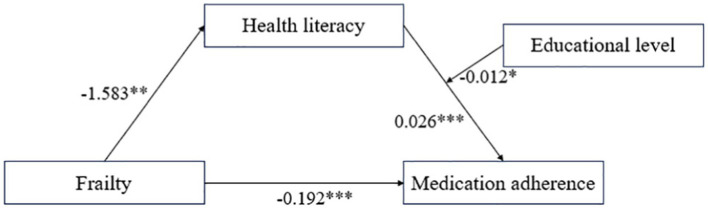
Path coefficient of the moderated mediation model. ^*^p < 0.05, ^**^p < 0.01, ^***^p < 0.001.

**Figure 2:**
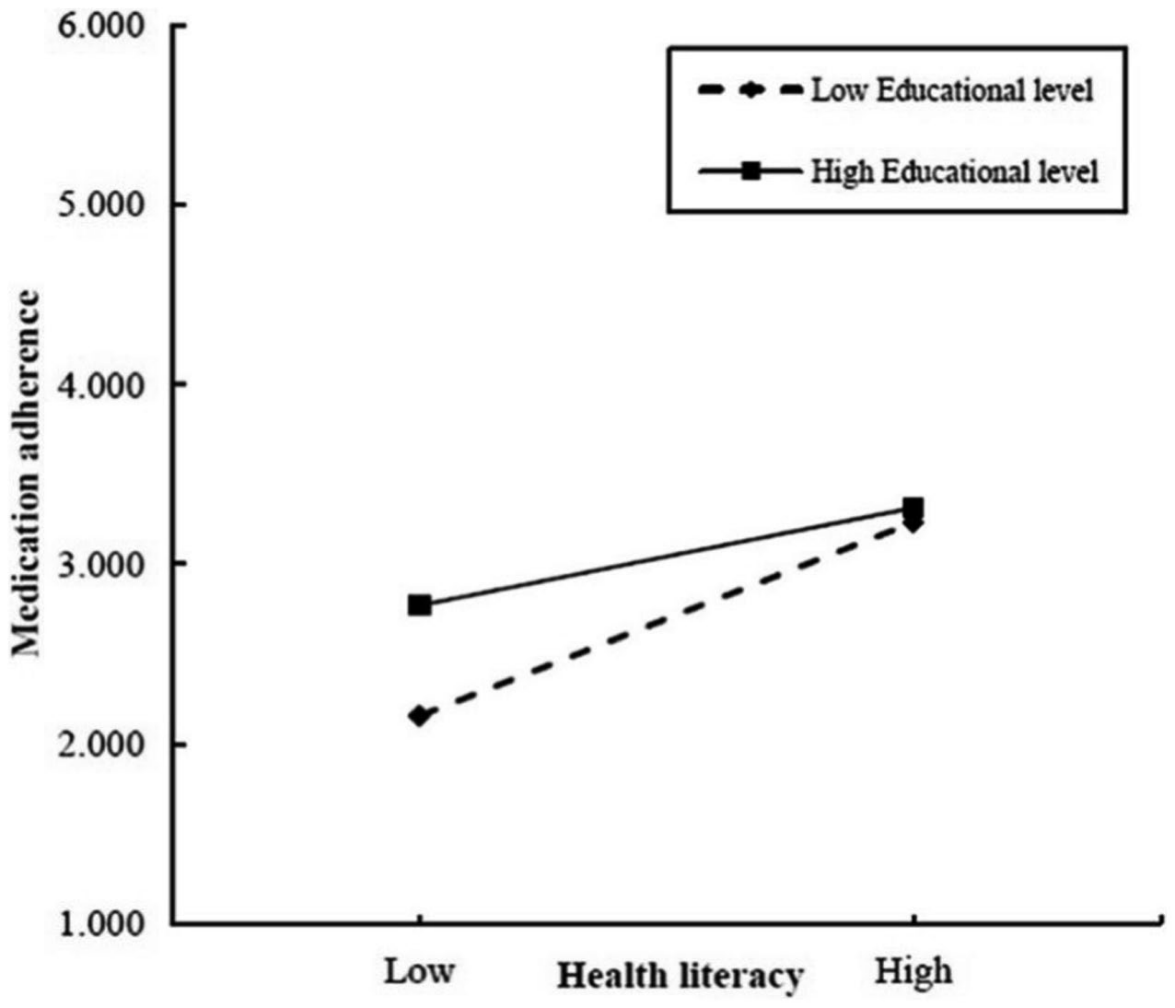
Simple slope test.

Educational level significantly moderated the indirect effect of frailty on medication adherence through health literacy (Tables 5, 6, Figure 1). Data reported in Table 5 show that health literacy was positively correlated with medication adherence (β = 0.026, *t* = 5.761, *p* < 0.001), and the interaction between health literacy and educational level exerted a significant negative effect on medication adherence (β = −0.012, *t* = −2.413, *p* < 0.05), while frailty had a significant negative effect on health literacy (β = −1.583, *t* = −2.833, *p* < 0.01). Furthermore, the direct effect of frailty on medication adherence was 0.192 (*t* = −4.116, *p* < 0.001). The path coefficients of the moderated mediation model are shown in Figure 1. The data revealed that the association between health literacy and medication adherence was stronger in individuals with low level(s) of education (β = 0.033, *t* = 5.953, *p* < 0.001) than in individuals with high levels of education (β = 0.017, *t* = 2.916, *p* < 0.01) (Figure 2). In addition, bias-corrected percentile bootstrap analysis confirmed that frailty indirectly influenced medication adherence through health literacy, which was moderated by different levels of education. Specifically, the conditional indirect effect between frailty and medication adherence was significant among individuals with all levels of education (low, effect −0.052 [95% CI −0.092 to −0.016]; medium, effect −0.041 [95% CI −0.071 to −0.012]; and high, effect −0.026 [95% CI −0.051 to −0.006]) (Table 6). The moderated mediation index was 0.019 (95% CI 0.003 to 0.043), confirming the effect of moderated mediation. In summary, these results confirm the second hypothesis. In other words, educational level moderated the relationship between health literacy and medication adherence. More specifically, health literacy had a significant positive predictive effect on medication adherence at different educational levels, and the positive predictive effect of health literacy on medication adherence gradually decreased with increasing educational level.

**Table 5:** Results of educational level moderate the mediation process.

	Model 1 (health literacy)		Model 2 (medication adherence)	
	*β*	*t*	*β*	*T*
Frailty	−1.583	−2.833^**^	−0.192	−4.116^***^
Health literacy			0.026	5.761^***^
Educational level			0.256	2.562^*^
Health literacy × Educational level			−0.012	−2.413^*^
*R* ^2^	0.061		0.203	
*F*	4.924^***^		12.087^***^	

Adjusted age, gender, BMI and hypertension complication.

^*^p < 0.05, ^**^p < 0.01, ^***^p < 0.001.

**Table 6:** Conditional indirect effect of educational level when health literacy mediated between frailty and medication adherence.

	Educational level	Effect	Boot SE	95% CI	
Health literacy	Min^#^	−0.052	0.020	−0.092	−0.016
	Mean	−0.041	0.015	−0.071	−0.012
	+1 SD	−0.026	0.011	−0.051	−0.006
Index of moderated mediation		0.019	0.010	0.003	0.043

Adjusted age, gender, BMI and hypertension complications.

^#^One SD below the mean is below the minimum observed in the data for educational level, so the minimum measurement on educational level is used for conditioning instead.

## Discussion

In the current study, 233 (60.1%) subjects demonstrated good medication adherence. According to previous research, the percentage of patients with good adherence fluctuates significantly (69–71). This may be due to the diverse evaluation methods and different demographic characteristics (14). Numerous studies have demonstrated the effects of frailty on medication adherence (30–32), although few have focused on the potential mediators and regulatory mechanisms that represent how and when frailty occurs. Our data demonstrated that frailty was related to medication adherence both directly and indirectly through health literacy. The educational level of older patients with hypertension may moderate health and, thus, influence medication adherence.

### Mediating role of health literacy

These results are consistent with previous studies reporting that frailty has a significant negative effect on health literacy (43, 58). The higher the frailty of the participant(s), the more their physiological capacity was reduced, which was associated with increased susceptibility to stressors, and the less likely they were able to obtain health-related information and, consequently, the lower their health literacy. Health literacy was a significant and positive predictor of treatment adherence, consistent with previous research findings (52, 53). People with low health literacy do not realize the importance of medication adherence, and they live an unhealthy lifestyle (15). Frailty had a negative and significant direct effect on medication adherence among older adult patients. Pharmacokinetics may partially explain this relationship. Due to changes in body fat lean tissue, and oxidative metabolic enzymes in frail patients, the distribution of drugs in the body and metabolism are prone to change, which predisposes to the occurrence of side effects and, in turn, poor medication adherence (30). Frailty in older adult patients is often associated with anxiety, depression, inadequate social support, lack of care, and supervision by others. When taking medication as prescribed by a physician, it is easier to exacerbate the adverse effects of taking the medication and even question the need to take the medication due to the lack of short-term efficacy, resulting in poor medication adherence (72). Health literacy is an inherent health promotion skill essential for processing medication information and taking medication correctly (73). Individuals with high health literacy are better able to receive, process, and understand health information; therefore, they participate more actively in decision-making and health actions, resulting in better medication adherence (74, 75). Inadequate health literacy is one of the main factors leading to poor medication adherence in patients with hypertension (46, 76). Therefore, improving health literacy has been suggested as an effective measure for improving medication adherence (76, 77).

### Moderating role of educational level

Data from the present study revealed that participant educational level moderated the indirect effect between frailty and medication adherence. The moderating point was in the second half of the intermediate chain; more specifically, the relationship between health literacy and medication adherence depended on educational level. Compared with individuals with higher education levels, the indirect effect was stronger for individuals with a lower education level (Table 6). Medication adherence among individuals with higher education levels was generally higher than that among those with lower education levels (Figure 2), suggesting that more attention should be devoted to individuals with lower educational levels. This moderation could be due to the fact that individuals with higher levels of education already have higher health literacy (78–81) as well as higher medication adherence (20, 82–84), which was consistent with our analysis of the association between educational level and health literacy (*r_s_* = 0.354, *p* < 0.001) and medication adherence (*r_s_* = 0.196, *p* < 0.001). However, for individuals with lower levels of education, an increase in health literacy could have a stronger impact on improving medication adherence. Individuals with higher levels of education tend to have higher health demands than those with lower levels of education and tend to understand the importance of medication (85). Conversely, those with lower levels of education are more likely to make mistakes, have less medical knowledge, and have superstitious beliefs (85). Therefore, they are more likely to make poor decisions when experiencing health problems. Improving educational level is difficult for older adults. However, features that benefit from education can also improve medication adherence by designing easy-to-understand educational materials, breaking down multistep or complex skills into smaller steps, and encouraging the older population to repeat information in their own words, such as reading, learning, and applying new information and specific skills (i.e., background knowledge) (86, 87). In addition, creating a positive community environment for trusting the science and rejecting superstitious beliefs would be helpful in improving medication adherence.

## Limitations and further research directions

Although the current study advances the understanding of the mediating and moderating mechanisms underlying the association between frailty and medication adherence among older adult individuals with hypertension, several limitations should be addressed. First, subjects were recruited from a single hospital, and the sample size was small; therefore, the results may not be generalizable to all older adult patients with hypertension. Further studies should be conducted in multicenter settings with sufficient sample sizes to ensure the generalizability of the conclusions. Second, this study used a cross-sectional design, which precluded the ability to infer causality. Longitudinal studies should be performed in the future to further verify the causal relationships between variables. Third, our data were self-reported, making them susceptible to a social desirability bias. Data for further studies should be obtained from objective tests or from multiple informants. Fourth, our study used a non-random sampling method, which has an unfavorable effect on the representativeness of the samples. We will use a more appropriate sampling method in the follow-up study.

## Conclusion

Although some studies have focused on the relationship between frailty and medication, little attention has been devoted to the mediation and moderation mechanisms underlying this association in older adult patients with hypertension. Our results suggest that more attention should be paid to patients with frailty and low educational level to achieve a greater breakthrough in medication adherence in older adult patients with hypertension. The clinical implication for us is that we should focus on hypertensive patients with frail, hypertensive patients with low health literacy, and hypertensive patients with low education level to better control the blood pressure of older adult patients with hypertension.

## Data availability statement

The raw data supporting the conclusions of this article will be made available by the authors, without undue reservation.

## Ethics statement

The studies involving humans were approved by Scientific Research and New Technology of Wannan Medical College Yijishan Hospital IRB. The studies were conducted in accordance with the local legislation and institutional requirements. The participants provided their written informed consent to participate in this study. Written informed consent was obtained from the individual(s) for the publication of any potentially identifiable images or data included in this article.

## Author contributions

AW: Data curation, Writing – original draft, Writing – review & editing. JW: Data curation, Investigation, Writing – original draft, Writing – review & editing. LZ: Data curation, Writing – review & editing. WC: Investigation, Writing – review & editing. LW: Writing – review & editing, Investigation. XT: Conceptualization, Supervision, Writing – review & editing. YJ: Conceptualization, Supervision, Writing – review & editing.
